# Unveiling the Molecular Mechanism of Intestinal Metabolite *para*‐Cresol in Modulating Neuroinflammation and Synaptic Dysfunction: Implications for Autism Spectrum Disorder

**DOI:** 10.1111/jnc.70457

**Published:** 2026-05-15

**Authors:** Wenjie Liao, Kristy Antonioni, Federica Silvestri, Monica Piemontese, Martina Bodria, Eleonora Daini, Antonio M. Persico, Michele Zoli, Andreas M. Grabrucker, Antonietta Vilella

**Affiliations:** ^1^ Department of Biomedical, Metabolic and Neural Sciences University of Modena and Reggio Emilia Modena Italy; ^2^ International School of Advanced Studies University of Camerino Camerino Italy; ^3^ Child & Adolescent Neuropsychiatry Program Modena University Hospital Modena Italy; ^4^ Department of Biological Sciences University of Limerick Limerick Ireland; ^5^ Bernal Institute University of Limerick Limerick Ireland; ^6^ Health Research Institute (HRI) University of Limerick Limerick Ireland

## Abstract

Autism spectrum disorder (ASD) is a diverse group of neurodevelopmental disorders that share similar behavioral patterns. Many individuals with ASD also exhibit gastrointestinal disturbances, likely linked to alterations in the composition and activity of intestinal bacteria, resulting in the overproduction and release of toxic metabolites into the systemic circulation. These toxins may reach the central nervous system (CNS) and activate microglia, which release inflammatory cytokines, ultimately impairing neuronal function. This microbiota‐gut‐brain axis has been suggested to play a crucial role in the pathogenesis of ASD. *Para*‐cresol (*p*‐Cresol) is one of these intestinal metabolites and could potentially contribute to ASD, as its urinary levels are elevated in autistic children under the age of 8 and correlate with symptom severity. Here, we aim to investigate the effect of *p*‐Cresol on various brain‐cell types to speculate on their specific contributions to ASD‐related synaptic dysfunctions. Immunocytochemistry assays revealed a significant decrease in excitatory (vesicular glutamate transporter VGLUT, postsynaptic density protein 95 PSD95) and inhibitory (vesicular GABA transporter VGAT) synaptic markers in *p*‐Cresol‐treated neurons during synaptogenesis. These effects were exacerbated in SH3 and multiple ankyrin repeat domains 3 (Shank3) knockdown neurons, which exhibit increased susceptibility of their synapses. *p*‐Cresol also induced a dose‐dependent inflammatory response in both astrocytes and microglia, characterized by the overexpression and release of inflammatory cytokines and chemokines such as interleukin 6 (IL6), interleukin 1β (IL1β), and C‐C‐ motif chemokine ligand 3 (CCL3). Our results suggest that *p*‐Cresol exerts cell‐specific effects on astrocytes and microglia, and the release of inflammatory cytokines and chemokines in response to *p*‐Cresol treatment may contribute to synaptic dysfunction, in addition to its direct effect on neurons.

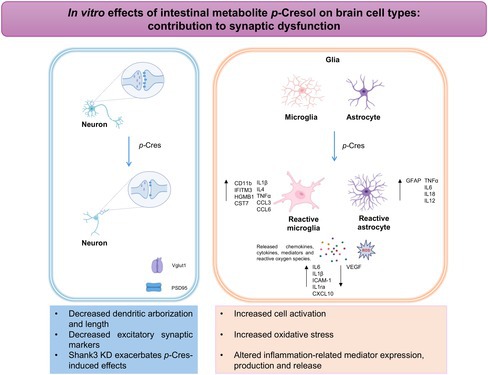

## Graphical abstract

Here we investigated the effect of *p*‐Cresol on different brain‐cell types to speculate on their specific contribution to autism spectrum disorder (ASD) synaptic dysfunctions. Our in vitro data show that *p*‐Cresol impairs dendritic arborization and length, disrupts the excitatory/inhibitory balance, and induces glial activation and inflammatory responses. All these data suggest that the release of inflammatory cytokines and chemokines upon *p*‐Cresol treatment might contribute to synaptic dysfunction, adding to the direct effect of *p*‐Cresol on neurons, thus contributing to ASD development.

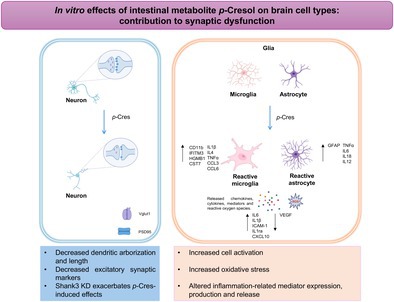

**Abbreviations**
ADHD: attention deficit/hyperactivity disorder
APOE: apolipoprotein E
ASD: autism spectrum disorder
CCL3: C‐C motif chemokine ligand 3
CCL6: C‐C motif chemokine ligand 6
CD11b: integrin αM
CINC‐1: cytokine‐induced neutrophil chemoattractant 1
CNS: central nervous system
CSF: cerebrospinal fluid
CST7: cystatin C
CXCL10: C‐X‐C motif chemokine ligand 10
DAPI: 4′,6‐diamidine‐2‐phenylindole dihydrochloride
DIV: day in vitro
DMEM: Dulbecco's modified eagle medium
E/I: excitation/inhibition
FBS: fetal bovine serum
GAPDH: glyceraldehyde‐3‐phosphate dehydrogenase
GFP: green fluorescent protein
hr: hour
ICAM‐1: intercellular adhesion molecule 1
IFITM3: interferon induced transmembrane protein 3
IL12: interleukin 12
IL18: interleukin 18
IL1β: interleukin 1β
IL4: interleukin 4
IL6: interleukin 6
LIX: LPS‐induced CXC chemokine
LTP: long term potentiation
MAP2: microtubule associated protein 2
MCP1: monocyte chemoattractant protein 1
min: minutes
NLRP1: NLR family pyrin domain containing 1
NLRP3: NLR family pyrin domain containing 3
O/N: overnight
P2Y12: purinergic receptor P2Y12
*p*‐Cresol: *Para*‐cresol
pCS: *Para*‐cresyl sulfate
Pen/Strep: penicillin/streptomycin
PFA: paraformaldehyde
PLL: poly‐L‐lysine
PSD95: postsynaptic density protein 95
ROS: reactive oxygen species
RPL27: ribosomal protein L27
RPMI: Roswell Park Memorial Institute
RPS29: ribosomal protein S29
RT: room temperature
Shank3: SH3 and multiple ankyrin repeat domains 3
STMN1: stathmin 1
TGFβ: transforming growth factor β
TIMP‐1: TIMP metallopeptidase inhibitor 1
TLR4: toll like receptor 4
TNFa: tumor necrosis factor α
VEGF: vascular endothelial growth factor
VGAT: vesicular GABA transporter
VGLUT: vesicular glutamate transporter

## 1 Introduction

Autism spectrum disorder (ASD) is a neurodevelopmental condition characterized by impaired social communication, restricted and repetitive/stereotypic behaviors, abnormal sensory processing, and cognitive dysfunction (Hodges et al. 2020). Epidemiological studies reported that approximately 1 in 100 children worldwide are affected by ASD, with the prevalence continuing to rise annually (Zeidan et al. 2022). ASD persists throughout life and may be accompanied by other conditions such as attention deficit/hyperactivity disorder (ADHD), intellectual disability, epilepsy, obsessive‐compulsive disorder, anxiety, mood disorders, sleep disorders, and gastrointestinal problems, including abdominal discomfort, chronic constipation, irritable bowel syndrome, and chronic diarrhea, and/or constipation (Zheng et al. 2022; Davoli‐Ferreira et al. 2021; Varghese et al. 2017).

Recent scientific research reveals a link between ASD and neuroinflammation. Accumulating data suggest that the microbiota‐gut‐brain axis exerts its action at least partially by modulating neuroinflammation, which contributes to a dysregulated immunomodulation of glial cells and/or abnormal synaptic development and function as potential drivers of ASD symptoms (Davoli‐Ferreira et al. 2021).

Metabolomics studies revealed increased levels of neuroactive uremic toxins, such as para‐cresol (*p*‐Cresol) and its metabolite *para‐cresyl sulfate* (pCS), in both urinary and fecal solutes of individuals with ASD compared to sex‐ and age‐matched controls (Pascucci et al. 2020; Bermudez‐Martin et al. 2021). Specifically, toxic *p*‐Cresol was significantly elevated in children with ASD younger than age 8, particularly in females, and regardless of sex, in more severely affected individuals (Altieri et al. 2011). Additionally, numerous studies have clearly demonstrated the existence of a close relationship between the composition of gut microbiota, gastrointestinal disorders, and ASD; in fact, fecal microbiota transplant therapy in ASD patients improved irritability, communication, and social skills and lowered fecal pCS to levels similar to those of neurotypical controls (Davoli‐Ferreira et al. 2021). Furthermore, animal studies showed that mice chronically exposed to *p*‐Cresol in drinking water demonstrate ASD‐like behaviors dependent on *p*‐Cresol‐induced changes in intestinal microbiota composition (Bermudez‐Martin et al. 2021).

Here, we speculate that gut physiology may affect neuronal functions not only directly but also indirectly, by impacting glial cells, key players in neuroinflammation. Notably, microglial cells ‐ the most abundant mononuclear phagocytes in the central nervous system (CNS) ‐ participate in neuronal physiological processes, such as synaptic pruning and the neuronal excitation/inhibition (E/I) ratio. Genome‐wide analysis of brain tissue from ASD individuals showed enrichment of markers related to activated microglia and expression of genes associated with immune and inflammatory gene ontology categories, compared to neurotypical controls, accompanied by changes in microglial morphology, density, and spatial localization (Wei et al. 2012). In addition, the presence of aberrant microglia and astrocyte immune activation well documented by neuropathological studies of *postmortem* human brain samples is compatible with the in vivo results of TSPO PET scanning studies (Zurcher et al. 2021) and of diffusion kurtosis imaging possibly linking to immune dysreactivity the reduced white matter integrity present in many ASD brains (Nagai et al. 2024). Regarding astrocytes' contribution, cytokine protein assays using brain tissue homogenates and cerebrospinal fluid (CSF) from ASD patients showed a significant increase of interleukin 6 (IL6), transforming growth factor β (TGFβ), and monocyte chemoattractant protein 1 (MCP1) (Vargas et al. 2005).

Moreover, IL6 regulates neuronal activity, and its downregulation alleviates abnormal gene expression patterns and impaired social communication in rodents (Wei et al. 2012, 2016). Elevated expression of IL6 in mouse brains is associated with impaired learning abilities, abnormal anxiety traits and habituations, and decreased social interactions (Wei et al. 2012; Gumusoglu et al. 2017). All these changes have been associated with an abnormal change in the shape, length, and distribution pattern of dendritic spines and the disrupted E/I balance (Wei et al. 2012; Gruol 2015). Of note, the treatment with dexmedetomidine, an α2 adrenergic receptor agonist, in BTBR mice, an idiopathic ASD mouse model, significantly reduces IL6 expression levels, improving ASD manifestations, except for repetitive behavior (Liang et al. 2024). Along with IL6, interleukin 1β (IL1β) and tumor necrosis factor α (TNFα) also contribute to synaptic activity regulating NMDA and AMPA receptor functions. Notably, microglia‐derived IL1β modulates the E/I balance in the hippocampus by suppressing GABAergic inhibitory transmission (Patterson 2015) and impairs synaptic function (Mishra et al. 2012), contributes to long‐term potentiation (LTP) inhibition and reduces synaptic strength, as well as modulates memory and learning under physiological conditions (Patterson 2015). Besides, IL1β level appears to be unchanged in the CNS of autistic individuals, but it is increased in their blood. Also, TNFα is critical in synaptic strength and plasticity via inhibitory LTP (Matta et al. 2019).

Although available data demonstrate a link between gut metabolites and brain dysfunction, the effect of *p*‐Cresol—the most abundant L‐tyrosine‐derived metabolite—on different brain‐cell types is currently poorly investigated, except for neurons (Guzman‐Salas et al. 2022). Here, we tested the effects of *p*‐Cresol exposure on neurons, astrocytes, and microglia to address their specific contribution to ASD‐related synaptic dysfunction and neuroinflammation.

## 2 Material and Methods

### 2.1 In Vitro Experiments

#### 2.1.1 Cell Culture

Three cell lines were used and cultured as follows. They are not listed as commonly misidentified cell lines by the International Cell Line Authentication Committee; all cell lines undergo authentication tests during the accessioning process and were not authenticated following purchase. DITNC1 (RRID:CVCL_0247, CRL‐2005, ATCC) rat astrocyte cells were cultured in Dulbecco's modified eagle medium (DMEM) (#D6546, Sigma) supplemented with 10% fetal bovine serum (FBS) (#2473339RP, Gibco), 2% L‐glutamine (#G7513, Sigma), and 1% penicillin/streptomycin (Pen/Strep) (P4333, Sigma). BV2 microglial cells (RRID:CVCL_0182) were cultured in Roswell Park Memorial Institute (RPMI) 1640 Medium (#12922006, Corning) supplemented with 10% FBS (#2473339RP, Gibco) and 1% Pen/Strep (#P4333, Sigma), or in DMEM (#D6546, Sigma), 10% FBS (#2473339RP, Gibco), 2% L‐glutamine (#G7513, Sigma), and 1% (Pen/Strep) (#P4333, Sigma). Both DITNC1 and BV2 have been validated to well recapitulate the molecular and functional properties of primary glial cells (Henn et al. 2009; Capuz et al. 2023). Only cells under passage 25 were used for experiments. Mixed rat hippocampal cells (#A36513, Thermo Fisher or #P10101, Innoscience Innoprot) were cultured in neurobasal medium (#21103‐049, Gibco), supplemented with 2% B27 supplement (#17504‐044, Gibco) to promote neuronal survival and differentiation, 1% Glutamax (#35050‐038, Gibco), and 1% Pen/Strep (#P4333, Sigma). Cells were maintained at 37°C in an atmosphere of 95% air and 5% CO_2_.

#### 2.1.2 *p*‐Cresol Treatment

A stock solution of 2000 μM *p*‐Cresol (#W233706, Sigma) was prepared using the complete medium of a specific cell line and then diluted according to each treatment.

#### 2.1.3 Lentiviral Knockdown of Shank3 in Rat Hippocampal Neurons

Rat hippocampal neural cells were seeded on coverslips coated with 0.05 mg/mL poly‐L‐lysine (PLL, #P6282, Sigma) in a 24‐well plate. Cells were transduced with green fluorescent protein (GFP)‐tagged SH3 and multiple ankyrin repeat domains 3 (Shank3) rat shRNA lentiviral particles (ORIGENE, #TL710469V) on *day* in vitro (DIV) 1, with a multiplicity of infection of 5 for 20 h (hr). The lentivirus‐containing medium was then replaced with fresh complete medium, and the cells were allowed to grow. On DIV 8, cells were treated with either vehicle or 100 μM *p*‐Cresol until DIV 14, followed by fixation and processing for immunofluorescent staining and confocal microscopy analysis, as described below. Specifically, Shank3 signal was measured only in GFP^+^/VGLUT^+^ and/or VGAT^+^ neurons.

#### 2.1.4 *P*‐Cres Treatment on Microglia and Astrocytes

DITNC1 and BV2 cells were seeded in a 0.05 mg/mL PLL‐coated 6‐well plate and grown overnight (O/N). The cells were treated with *p*‐Cresol at concentrations of 5, 50, 150, and 300 μM for 24 h. On day 2, the medium was collected for protein release assays, followed by one wash in PBS, after which RNA was extracted according to the manufacturer's instructions for the RNeasy Mini Kit (#74104, Qiagen).

#### 2.1.5 Immunofluorescence Staining

DIV14 neurons were washed with PBS after removing the cell medium and fixed with 4% paraformaldehyde (PFA) for 20 min (min) at room temperature (RT). Subsequently, the cells were washed with PBS three times, 5 min each at RT. The cells were incubated with blocking solution (10% FBS in PBS) for 1 h at RT to avoid unspecific binding of antibodies. Afterward, the cells were incubated with the primary antibody (see Table 1) in blocking solution at 4°C O/N, followed by 3 washing steps in PBS to wash out the unbound primary antibody. The appropriate secondary antibody was diluted in blocking solution and applied for 1 h at RT. After 3 washes in PBS, the cells were stained with 4′,6‐diamidine‐2‐phenylindole dihydrochloride (DAPI) for 5 min and mounted with VectaMount AQ (#H‐5501, Vector Laboratories) on glass slides.

**TABLE 1:** List of primary and secondary antibodies.

Target	Host species	Dilution	Company, catalog number
MAP2	Chicken	1:1000	Millipore, #AB15452
PSD95	Rabbit	1:200	Sigma ‐ Merck, #ZRB1257
VGAT	Rabbit	1:200	Synaptic system, #131003
VGLUT	Guinea pig	1:1000	Synaptic system, #135304
Shank3	Guinea pig	1:500	Asciks, produced at home
GFP	Mouse	1:1000	Millipore, #MAB3580
Anti‐mouse 488	Goat	1:500	Invitrogen, #A11001
Anti‐guinea pig 488	Goat	1:500	Invitrogen, #A11073
Anti‐rabbit 488	Goat	1:500	Invitrogen, #A11008
Anti‐guinea pig 568	Goat	1:500	Invitrogen, #A11075
Anti‐rabbit 568	Goat	1:500	Invitrogen, #A11011
Anti‐chicken 647	Goat	1:500	Invitrogen, #A21449

#### 2.1.6 RNA Extraction and Quantitative Polymerase Chain Reaction (qPCR)

DITNC1 and BV2 cells were incubated with *p*‐Cresol at 50, 150, and 300 μM concentrations. After 24 h, media were removed, cells were washed in PBS and collected after trypsinization (4 min at 37°) and centrifugation (5 min at RT, 300 g). The cell pellets were lysed on ice, and RNA extraction was performed according to the manufacturer of the RNeasy Mini kit (#74104, Qiagen). Isolated mRNA was reverse transcribed to cDNA using random hexamers and M − MLV Reverse Transcriptase (Promega Corporation) following the instructions provided by the manufacturer. Samples were heated at 70°C for 5 min to eliminate any secondary structures, then incubated at 23°C for 10 min, 1 h at 37°C, and 5 min at 95°C before being chilled at 4°C using a thermocycler T Gradient (SimpliAmp, Applied Biosystem). The amount of cDNA was quantified with iTaq Universal SYBR Green Supermix (#1725125, Bio‐Rad) using a Bio‐Rad RT‐PCR iCycler. Each PCR reaction was performed in triplicate using 300 nM of each primer (Table 2), 10 μL of iTaq Universal SYBR Green Supermix (Bio‐Rad), cDNA and nuclease‐free water with the following cycling parameters: 2 min at 95°C and 40 cycles of 5 min at 95°C and 30 s at 60°C, followed by 5 s at 95°C and 65°C to 95°C melting curve analysis.

**TABLE 2:** List of primers used for Real‐Time PCR analyses.

Function	Species	Transcript	NBCI Ref Seq	Primer	Primary sequence (5′–3′)
Housekeeping	Mouse	RPL27	NM_011289	Fw	ACATTGACGATGGCACCTC
				Rv	GCTTGGCGATCTTCTTCTTG
	Mouse	RPS29	NM_009093	Fw	TGAAGGCAAGATGGGTCAC
				Rv	GCACATGTTCAGCCCGTATT
	Rat	GAPDH	NM_017008	Fw	CAAGGTCATCCATGACAACTTTG
				Rv	GGGCCATCCACAGTCTTCTG
	Rat	APOE	NM_00127068	Fw	TTGGTCCCATTGCTGACAGG
				Rv	GCGCAGGTAATCCCAGAAGC
Microglial	Mouse	CD11b	NM_00108296	Fw	TACCGTCTACTACCCATCTGGC
				Rv	TTGGTGAGCGGGTTCTGG
	Mouse	IFITM3	NM_025378	Fw	CCGTGAAGTCTAGGGATCGGA
				Rv	GTGTGAAGGTTTTGAGCGTT
	Mouse	CST7	NM_009977	Fw	CAGGAAGACCATGCATCACCA
				Rv	ATAGAGTCCGCTTCAAGGCAG
	Mouse	P2Y12	NM_00135701	Fw	CATTGCTGTACACCGTCCTG
				Rv	AACTTGGCACACCAAGGTTC
	Mouse	STMN1	NM_019641	Fw	AGGTGCTCCAGAAAGCCATC
				Rv	TCCACGTGCTTGTCCTTCTC
Inflammatory response	Mouse	NLRP1	NM_00100414	Fw	GGTGTGCTGGTTGGTCTGC
				Rv	GTGCTGTGGTGGTCTGTGAG
	Mouse	NLRP3	NM_145827	Fw	GCTCCAACCATTCTCTGACC
				Rv	AAGTAAGGCCGGAATTCACC
	Mouse	TNFα	NM_013693	Fw	GGTTCTGTCCCTTTCACTCAC
				Rv	TGCCTCTTCTGCCAGTTCC
	Rat	TNFα	NM_012675	Fw	CCACCACGCTCTTCTGTCTA
				Rv	TGATCTGAGTGTGAGGGTCTG
	Mouse	IL6	NM_031168	Fw	ACCGCTATGAAGTTCCTCTC
				Rv	CTCTGTGAAGTCTCCTCTCC
	Rat	IL6	NM_012589	Fw	CTTCACAAGTCGGAGGCTTAAT
				Rv	ATGAACAGCGATGATGCACT
	Mouse	IL1β	NM_008361	Fw	GCTTCAGGCAGGCAGTATC
				Rv	AGGATGGGCTCTTCTTCAAAG
Immunological	Mouse	IL4	NM_021283	Fw	CGGCACAGAGCTATTGATGG
				Rv	CATCCGTGGATATGGCTCCT
	Rat	IL4	NM_201270	Fw	CCCCCACCTTGCTGTCACCCTG
				Rv	CACCCTGGAAGCCCTGCAGATGA
	Mouse	IL18	NM 008360	Fw	ACCAAGTTCTCTTCGTTGAC
				Rv	TCACAGCCAGTCCTCTTAC
	Rat	IL18	NM_019165	Fw	ACCGCAGTAATACGGAGCAT
				Rv	CAGTCTGGTCTGGGATTCGT
	Mouse	TLR4	NM_021297	Fw	TTCACCTCTGCCTTCACTAC
				Rv	CACTACCACAATAACCTTCCG
	Rat	TLR4	NM_019178	Fw	TCAGTGTGCTTGTGGTAGCC
				Rv	CTCGTTTCTCACCCAGTCCT
	Mouse	CXCL10	NM_021274	Fw	TTCTGCCTCATCCTGCTG
				Rv	AGACATCTCTGCTCATCATTC
	Mouse	CCL3	NM_011337	Fw	CATATGGAGCTGACACCCCG
				Rv	TCTTCCGGCTGTAGGAGAAGC
	Mouse	CCL6	NM_009139	Fw	CTGGCCTCATACAAGAAATGGA
				Rv	TTGGAGGGTTATAGCGACGAT
	Rat	IL12	NM_022611	Fw	CCACGGACGACATGGTGAGG
				Rv	AGTGTGCTGGTTTTGTCCCGT

### 2.2 Migration Assay

DITNC1 and BV2 cells were seeded on a 0.05 mg/mL PLL‐coated 6‐well plate and grown O/N to reach 90% confluency. To assess their migration ability, a scratch was created with a p200 pipette tip in the middle of the plate, followed by medium removal and one wash in PBS and fresh medium adding. Then, cells were incubated with *p‐*Cresol at concentrations of 50 μM, 150 μM, and 300 μM and imaged at 6, 24, and 48 h with an optical microscope at 4×; qualitative analyses were performed to have an indicative assessment of *p*‐Cresol treatment on the migration ability and/or viability of the cells.

### 2.3 CellROX For Oxidative Stress Detection

DITNC1 and BV2 cells were seeded on a 0.05 mg/mL PLL‐coated Greiner multiwell dish and grown overnight. The cells were incubated with *p‐*Cresol at concentrations of 50 μM, 150 μM, and 300 μM for 24 h. Untreated wells served as the negative control. After 24 h, the cells were live stained with 5 μM CellROX green dye (#C10444, ThermoFisher) for 30 min at 37° with 5% CO_2_. Subsequently, the cells were washed twice with PBS, fixed with 4% PFA for 15 min in the dark at RT, and washed three times with PBS. The nuclei were then stained with DAPI, followed by two washes in deionized water, and the cells were imaged in PBS using the ImageXpress Microscope (Molecular Devices).

### 2.4 Proteome Profiler Kit

Produced/released cytokine mapping in cell lysates and/or media collected from astrocytes and microglia after 24 h treatment was performed using a cytokine array assay, separately (for astrocytes) or as a mix with a proteome profiler kit Rat Cytokine Array Kit (#ARY008, RND Systems) according to the manufacturer's instructions. For mixed preparations, cell lysates or medium from separately cultured astrocytes and microglia were collected and then mixed for proteome profiling.

### 2.5 Enzyme‐Linked Immunosorbent Assay (ELISA)

The concentration of IL6 protein in collected media was detected using rat and mouse IL6 ELISA kits (#R600B and #M600B, R&D, Systems, respectively) according to the manufacturer's instructions.

### 2.6 Image and Statistical Analyses

Immunolabeled slides were visualized and imaged with Leica confocal microscope SP8 or ImageXpress microscope (Molecular Devices) at 40× and analyzed with ImageJ software.

In neuron experiments, three independent cell culture preparations were quantified; at least 10 neurons per slide were analyzed for each condition. For each neuron, at least three dendrites were used for puncta quantification.

Total length, number of primary and secondary dendrites were manually measured from each neuron.

For synaptic puncta quantification, neurons successfully transduced with lentiviral particles were identified based on GFP positivity. For each coverslip, GFP‐positive neurons were selected and Shank3 immunoreactivity (IR) was quantified by measuring both the number of puncta and mean fluorescence intensity in Scr and KD conditions. At least 10 neurons per coverslip were analyzed. Dead or morphologically compromised neurons, as well as densely clustered regions where accurate quantification was not possible, were excluded from the analysis. Images were converted to 16‐bit, appropriate thresholds were applied to each antibody, and only puncta within 5–70 or 5–100 pixels^2 of size were selected, 6.52 pixels = 1 μm.

In glial experiments, three independent cell culture preparations were quantified; at least 8 fields of view were analyzed for each condition.

For glial cell viability, DAPI+ cells were both manually and automatically counted. In automatic counting, images were converted to 16‐bit, an appropriate threshold was applied to identify single cells, and 50‐infinity pixel^2^ was selected as size, 3.52 pixels = 1 μm; the results were further confirmed by manual counting. In microglial cells, DAPI+ nuclei with proliferating morphology were manually counted to assess the proliferation ratio (Talaat et al. 2024).

For CellROX analysis, cells were imaged in a 24‐well plate with ImageXpress microscope (Molecular Devices) at 20×; images were then converted to 16‐bit with ImageJ, and total GFP+ area was quantified after applying appropriate threshold; such area was then divided by the number of DAPI+ cells (counted as previously described) in the same field to assess average GFP+ area per cell, then expressed in % of Ctrl group.

For proteome profiling, blot images were taken with Q9 Alliance, Uvitec, and cytokine spot densities were quantified with ImageJ. Mean gray value was calculated from every single spot, delimitated with freehand selection, adjusted according to reference spots of each experimental group, then expressed in % of Ctrl group.

Data are presented as mean ± standard error of the mean (SEM). Normal data distribution was confirmed by Shapiro–Wilk tests; accordingly, group differences were analyzed using two‐way ANOVA or repeated measures ANOVA (treatment and experiment as factors), as appropriate. Student's *t*‐test and one‐way ANOVA were performed in released medium analysis (ELISA and proteome profiling). No outliers were excluded from the analyses.

Statistical analyses were conducted using SPSS software (version 26), with *p* < 0.05 considered significant (*p* < 0.05 *; *p* < 0.01 **; *p* < 0.001 ***) and 0.10 > *p* > 0.05 indicating a trend toward significance (#).

## 3 Results

### 3.1 *p*‐Cresol Treatment Reduced Dendrites Arborization and Length and Synapse Number in Rat Hippocampal Neurons

**FIGURE 1:**
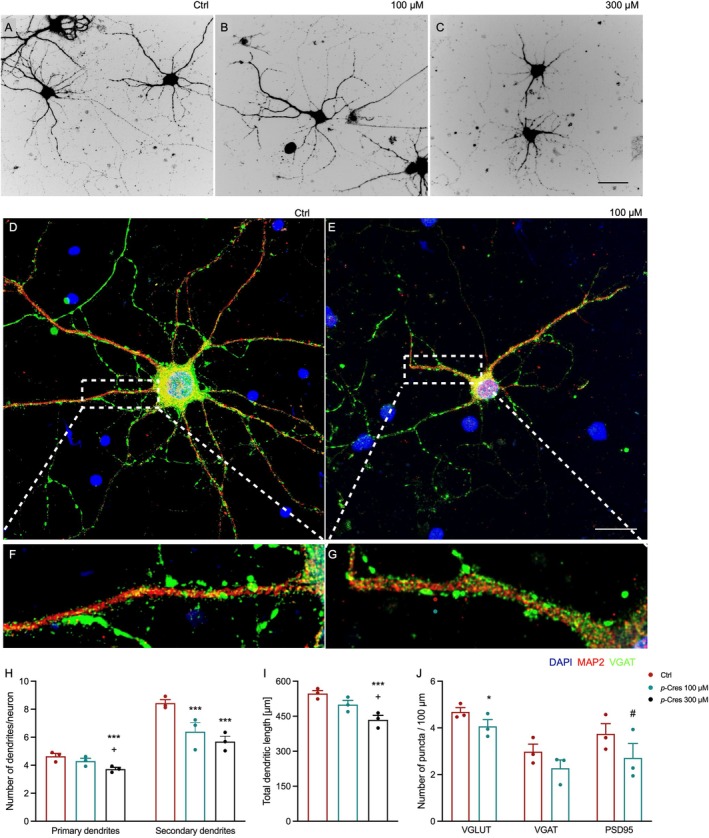
Effects of *p‐*Cresol on rat hippocampal neurons. (A–C) Primary and secondary dendritic arborization during synaptogenesis: Representative images of rat hippocampal MAP2^+^ neurons (dark) incubated with vehicle (control, Ctrl), 100 or 300 μM of *p*‐Cresol from DIV8 to DIV14. Scale bar = 20 μm. (D–G) Post‐synaptic density in rat hippocampal neurons: Representative images of rat hippocampal MAP2^+^ (red) and VGAT^+^ (green) neurons incubated with vehicle or 100 μM of *p*‐Cresol from DIV8 to DIV14. Cell nuclei were counterstained with DAPI (blue). Scale bar = 20 μm. (H) Number of primary and secondary dendrites/neuron, (I) total dendritic length, and (J) number of VGLUT^+^, VGAT^+^, and PSD95^+^ puncta/100 μm dendrite. All data were expressed as mean ± SEM values and analyzed by two‐way ANOVA followed by Tukey's post hoc comparisons: *p < 0.05, ***p < 0.001, ****p < 0.0001, #0.05 < *p* < 0.1 vs. ctrl; +*p* < 0.05 vs. 100 μM.

Neuronal morphology and synaptic organization were assessed using both brightfield imaging and immunofluorescence analysis. Brightfield images (Figure 1A–C) show representative neurons with well‐defined somata and extensive dendritic arborization in Ctrl condition, and a dose‐dependent loss of fine cytoarchitecture in *p*‐Cresol conditions. Neurons exhibit typical multipolar morphology, with multiple primary dendrites emerging from the soma and branching into secondary processes. Immunofluorescence labeling (Figure 1D,E) reveals detailed neuronal structure and synaptic distribution. MAP2 (red) staining highlights dendritic processes, while VGAT (green) staining marks inhibitory presynaptic puncta. DAPI (blue) labels nuclei. High‐magnification views (Figure 1F,G) demonstrate punctate VGAT signal localized along MAP2‐positive dendrites, indicating synaptic contacts in both Ctrl and after *p*‐Cresol treatment. Quantitative analysis revealed that both 100 μM and 300 μM *p*‐Cresol concentrations reduced secondary dendrite number and, at higher concentrations, also primary dendrite number (Figure 1H), accompanied by decreases in dendrite length (Figure 1I). The dendrite number decrease was correlated with synaptic density changes, shown by a significant reduction in excitatory presynaptic marker vesicular glutamate transporter (VGLUT) and a trend toward statistical reduction in postsynaptic marker post‐synaptic density protein 95 (PSD95)^+^ puncta 6 days after 100 μM *p*‐Cresol treatment. The number of inhibitory presynaptic marker vesicular GABA transporter (VGAT)^+^ puncta was unaffected (Figure 1J).

Of note, the number of MAP2^+^ cells remains unaffected, regardless of *p*‐Cresol concentration (Figure S1).

Overall, these results indicate that the experimental condition leads to reduced dendritic complexity and altered synaptic organization, suggesting impaired neuronal connectivity.

### 3.2 *p*‐Cresol‐Induced Effects Were Exacerbated by Shank3 Knockdown in Rat Hippocampal Neurons

**FIGURE 2:**
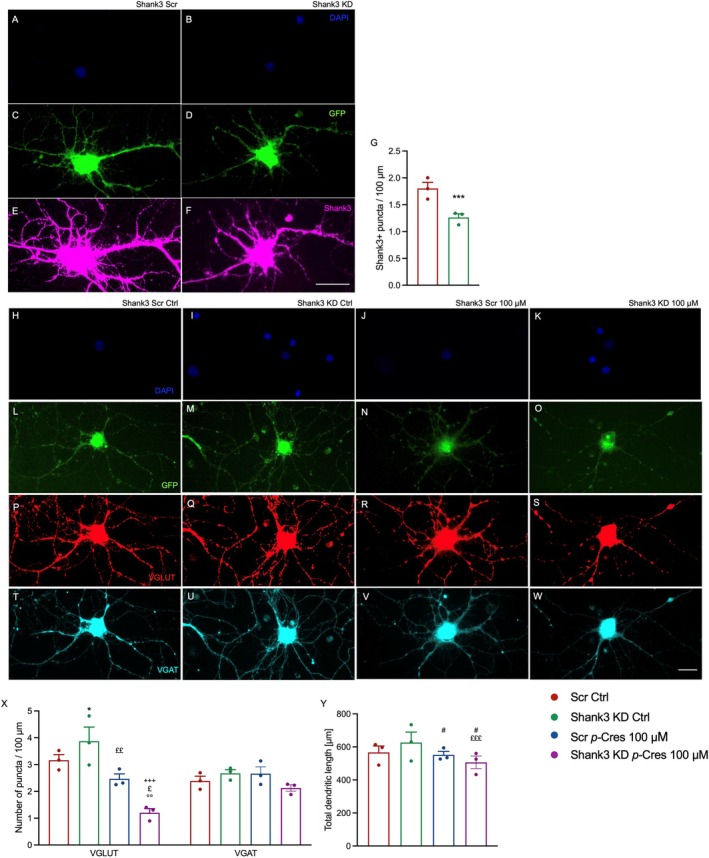
Shank3 knockdown in rat hippocampal neurons. Hippocampal neurons were transduced with GFP‐tagged Shank3 lentiviral shRNA particles on DIV1, then treated with vehicle or 100 μM *p*‐Cresol from DIV8 to DIV14. Cells were immunostained with anti‐GFP, ‐Shank3, ‐VGLUT, and ‐VGAT Abs at the end of treatment. (A–F) Representative images of GFP^+^ neurons (green) stained with anti‐Shank3 (magenta) in Scr (full expression of Shank3) and KD conditions (reduced expression of Shank3). Cell nuclei were counterstained with DAPI (blue). Scale bar = 20 μm. (G) Transfection efficiency of Shank3 KD lentiviral particles expressed as Shank3 puncta/100 μm. (H–W) Representative images of GFP^+^ (green) Shank3 Scr and KD neurons stained with VGLUT (red) and VGAT (cyan) in ctrl and 100 μM *p*‐Cresol conditions. Cell nuclei were counterstained with DAPI (blue). Scale bar = 20 μm. (X) VGLUT^+^ and VGAT^+^ puncta / 100 μm in Scr and Shank3 KD conditions, treated with vehicle or 100 μM *p*‐Cresol. (Y) Effect of Shank3 KD combined with *p*‐Cresol treatment on neuronal dendrite length (μm). All data were expressed as mean ± SEM values and analyzed by two‐way ANOVA followed by Tukey's post hoc comparisons: *p < 0.05; **p < 0.01; ***p < 0.001 vs. Scr ctrl; £*p* < 0.05; ££*p* < 0.01; £££*p* < 0.001 vs. Shank3 KD Ctrl; +++*p* < 0.001 vs. Scr *p*‐Cres 100 μM; #0.10 > *p* > 0.05.

Shank3^+^ puncta quantification per dendrite in DAPI^+^/GFP^+^ neuronal‐like cells (with neuronal morphology) showed that the lentiviral particles successfully silenced (by about 30%) Shank3 proteins (Figure 2A). Count of VGLUT^+^ and VGAT^+^ puncta (Figure 2B) and measurement of total dendrite length (Figure 2C) showed a reduction in VGLUT^+^ but not in VGAT^+^ puncta after *p*‐Cresol treatment, an effect that was enhanced by Shank3 knockdown (KD) compared to the scrambled (Scr) group.

With regard to dendrite length, a significant treatment*Shank3 KD interaction was detected, indicating that *p*‐Cresol treatment becomes effective only in Shank3 KD neurons. Shank3 KD alone did not change synaptic integrity or dendrite length.

### 3.3 *p*‐Cresol Treatment Caused Cell‐Specific and Dose‐Dependent Damage and Oxidative Stress

In the migration assay (see Figure S2), qualitative analysis showed a cell‐specific response to *p*‐Cresol: microglia started to migrate toward the gap from 6 h (Figure S2A.1–D.1) at all concentrations, filling the gap at 48 h (Figure 2A.3–D.3). Importantly, at the highest concentration, the cells were less compared to other groups. As regards astrocytes, they proliferated around the gap for the first 24 h (Figure 2E.1–H.2,E.2–H.2) without showing noticeable migratory ability; at 48 h, the cells completely covered the well (Figure 2E.3–H.3).

**FIGURE 3:**
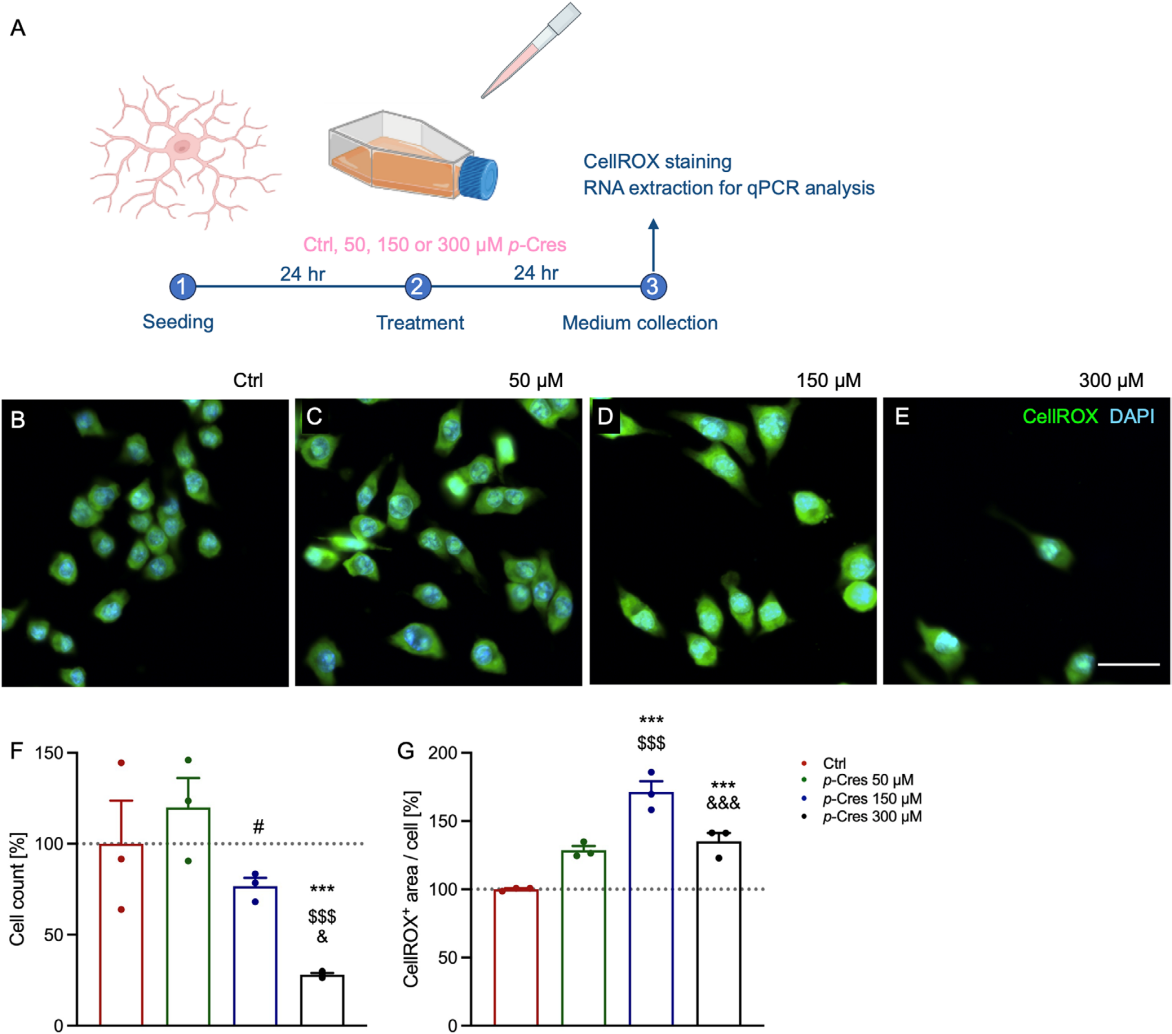
Effects of *p*‐Cresol exposure on BV2 microglial cells. (A) Experimental workflow: (B–E) Representative images of microglial cells incubated with the vehicle (Ctrl), 50, 150, or 300 μM of *p*‐Cresol for 24 h (B‐E, respectively) following CellROX (green) staining. Cell nuclei were counterstained with DAPI (blue). Scale bar = 20 μm. (F) Quantitative results of DAPI^+^ microglial cell counts 24 h after stimulation with the vehicle or different *p*‐Cresol concentrations (*n* = 3 independent experiments). (G) Effects of *p‐*Cresol on oxidative stress in BV2 cells assessed using CellROX staining. Results are expressed as mean ± SEM values. Statistical analyses were conducted using two‐way ANOVA with Tukey's multiple comparison tests; *p < 0.05 vs. Ctrl; &*p* < 0.05 vs. *p‐*Cresol 150 μM; ***p < 0.001 vs. Ctrl; $$$*p* < 0.001 vs. *p‐*Cresol 50 μM; &&&*p* < 0.001 vs. *p‐*Cresol 150 μM; #0.10 > *p* > 0.05.

To phenotypically characterize the glia‐specific response to *p‐*Cresol exposure, BV2 microglial cells were treated with vehicle (Ctrl), 50, 150, or 300 μM *p*‐Cresol for 24 h, and subsequently stained with CellROX green dye to label reactive oxygen species (ROS) production (Figure 3). Counting of DAPI^+^ cells showed that *p*‐Cresol affected microglia viability (Figure 3F), with a 74% reduction at the highest concentration. Concerning ROS production (Figure 3G), we found a dose‐dependent increase up to 150 μM, then a decrease at 300 μM *p*‐Cresol concentration.

**FIGURE 4:**
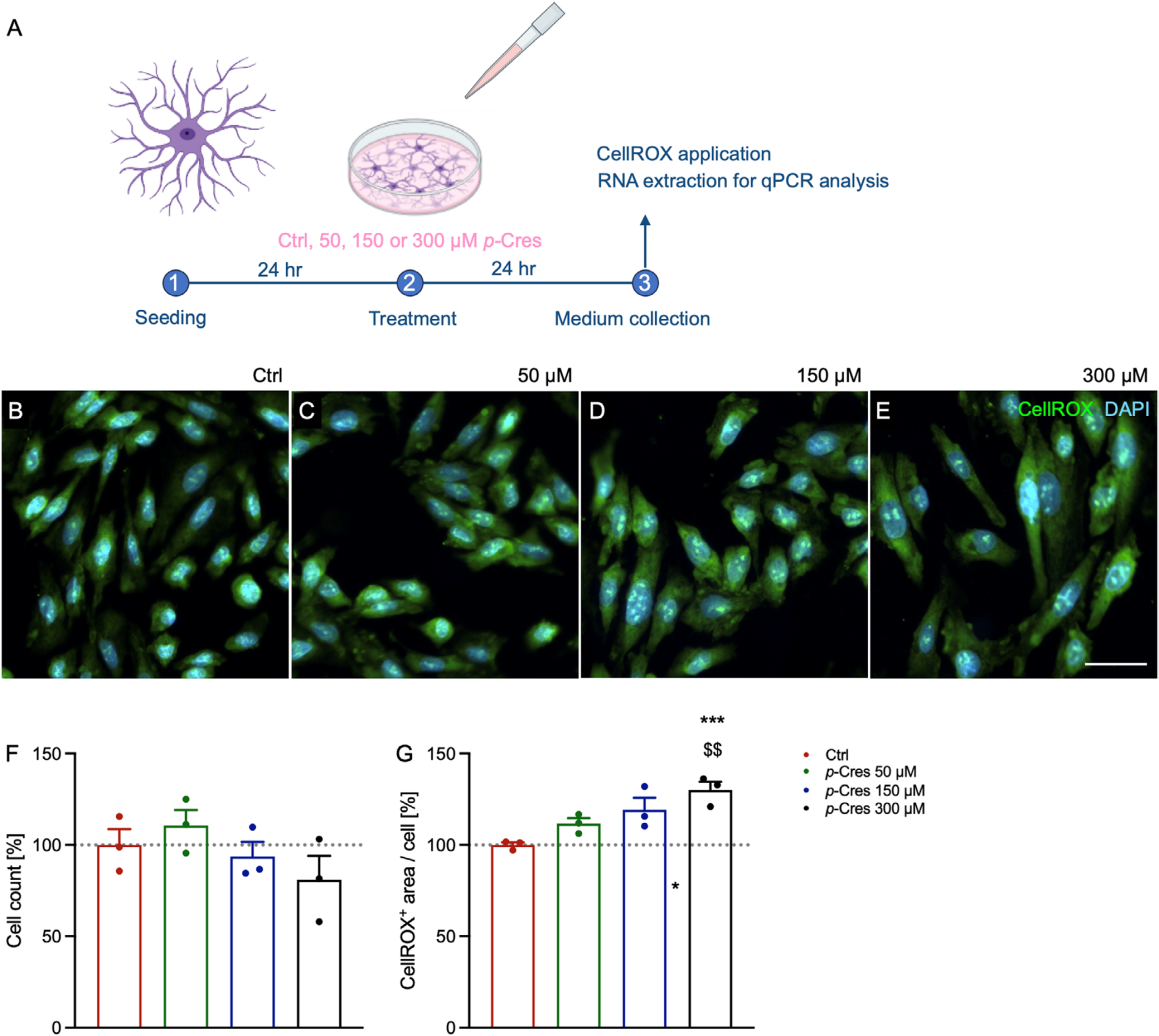
Effects of *p‐*Cresol on DITNC1 astrocytes. (A) Experimental workflow: (B–E) Representative images of astroglial cells incubated with vehicle (Ctrl), and concentrations of 50, 150, or 300 μM of *p*‐Cresol for 24 h following CellROX (green) staining. Cell nuclei were counterstained with DAPI (blue). Scale bar = 20 μm. (F) Quantitative results of the DAPI^+^ astrocyte cell count 24 h after stimulation with either vehicle or different *p*‐Cresol concentrations. (G) Effects of *p‐*Cresol on oxidative stress in DITNC1 cells measured using CellROX staining. Results are expressed as mean ± SEM values. Statistical analyses were conducted using two‐way ANOVA with Tukey's multiple comparison tests: *p < 0.05 vs. Ctrl, ***p < 0.001 vs. Ctrl, $$*p* < 0.01 vs. *p‐*Cresol 50 μM.

In contrast to BV2 microglia, DITNC1 cells did not exhibit a significant reduction in cell number under any experimental conditions (Figure 4F). In terms of ROS production, the response was dose‐dependent, with the highest fluorescence levels corresponding to the highest treatment concentration (Figure 4G).

Finally, to determine how cells recovered from the application of *p*‐Cresol 24 h after its removal, microglial cells were stained with DAPI. From counting of viable DAPI^+^ cells, we observed a 15% decrease in the number of BV2 cells treated with *p*‐Cresol compared to the Ctrl group (Figure 3A), whereas the count of proliferating cells showed that the two groups have the same proliferation ratio (Figure 3B).

### 3.4 *p*‐Cresol Treatment Induced Cell‐Specific and Dose‐Dependent Inflammatory Responses

**FIGURE 5:**
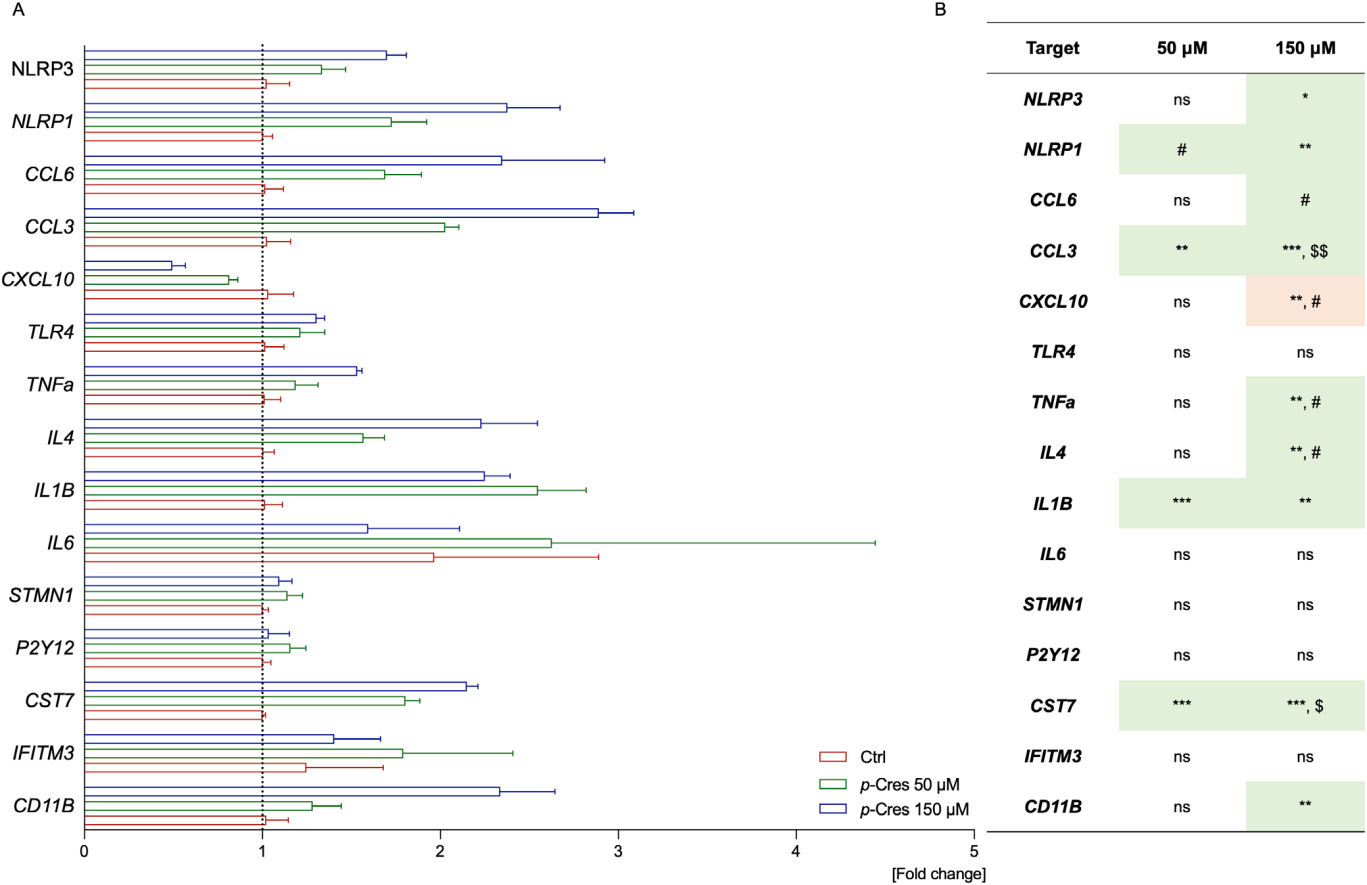
Effect of *p*‐Cresol on mRNA expression of BV2 microglial cells. BV2 cells were treated with vehicle (Ctrl), 50 or 150 μM of *p‐*Cresol for 24 h, then the total RNA was extracted and reverse transcribed, and qPCR was conducted. (A) Data are presented as fold change normalized over the Ctrl group mean value and calibrated over RPS29/RPL27 housekeeping genes. The experimental conditions were tested in quadruplicate; data were expressed as mean ± SEM values and analyzed by one‐way ANOVA. (B) The colorimetric scale was used to show up‐ (green) or down‐ (red) regulated genes; *p < 0.05 vs. Ctrl; **p < 0.01 vs. Ctrl; ***p < 0.001 vs. Ctrl; $*p* < 0.05 vs. *p‐*Cresol 50 μM; $$*p* < 0.01 vs. *p‐*Cresol 50 μM; #0.10 > *p* > 0.05.

To assess the inflammatory response of glial cells, the gene expression of inflammatory response‐related mediators was evaluated in BV2 microglia treated for 24 h with Ctrl, 50, or 150 μM *p*‐Cresol (Figure 5), as well as 24 h after *p*‐Cresol removal (see Figure S4). Genes associated with the functional status of microglia (e.g., CD11b, CST7) were upregulated after 24 h of treatment but decreased following *p*‐Cresol removal; a similar trend was observed in cytokines (IL1B, IL4, TNFa) and chemokines (C‐C motif chemokine ligand 3 and 6, CCL3, CCL6). In general, the 24‐h treatment induced upregulation of these genes, whose expression returned to baseline or was downregulated after *p*‐Cresol removal.

**FIGURE 6:**
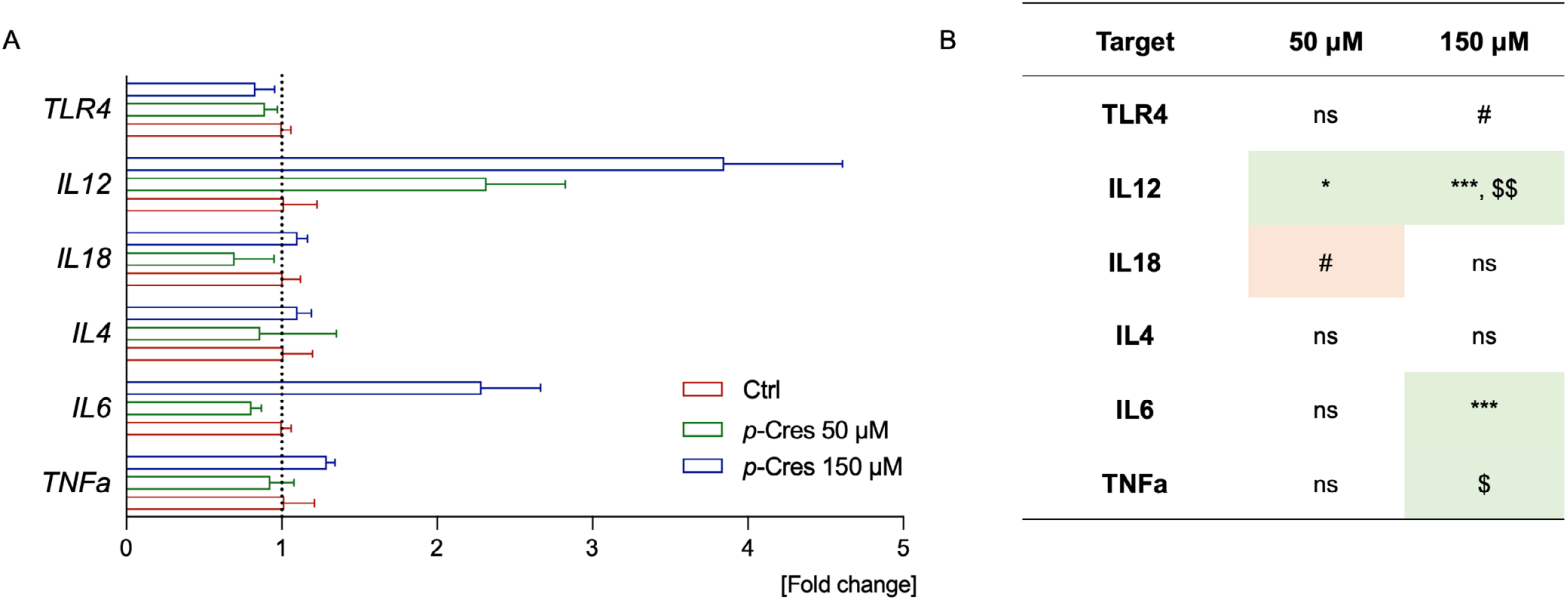
Effects of *p*‐Cresol on gene expression of DITNC1 astrocytes. DITNC1 cells were treated with vehicle, 50 or 150 μM of *p‐*Cresol for 24 h, then the total RNA was extracted, and qPCR was conducted. (A) Data are presented as fold change normalized over the Ctrl group mean value and calibrated over GAPDH/APOE housekeeping genes. The experimental conditions were tested in triplicate, and data were expressed as mean ± SEM values and analyzed by one‐way ANOVA (B). The colorimetric scale was used to show up‐ (green) or down‐ (red) regulated genes; *p < 0.05; ***p < 0.001; $$*p* < 0.01 vs. *p‐*Cresol 50 μM; #0.10 > *p* > 0.05.

In DITNC1 astrocytes (Figure 6), the upregulation of certain genes persisted after the removal of p‐Cresol (e.g., TNFa, IL6, IL12) (see Figure S5), indicating a different response kinetics in this cell type compared to microglia.

**FIGURE 7:**
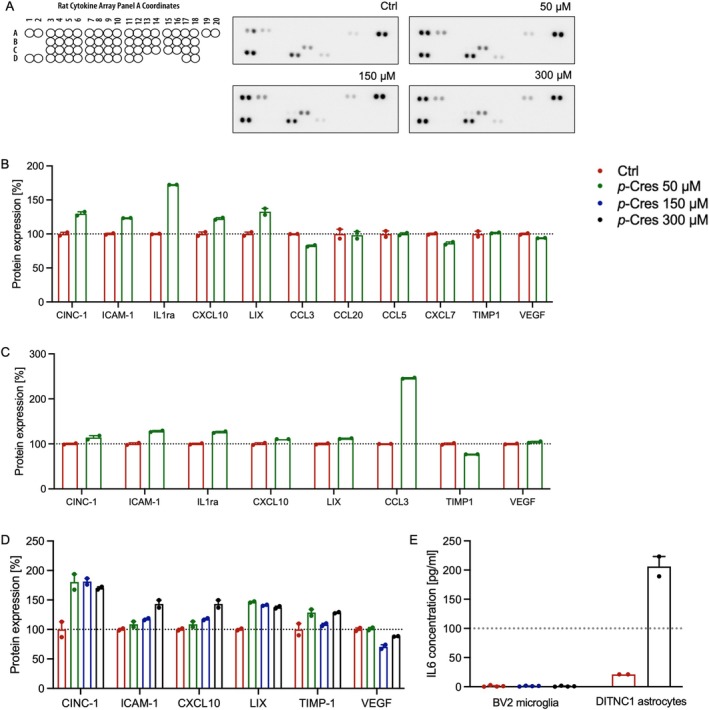
Production and release of inflammation‐related proteins after *p*‐Cresol treatment. (A) Representative dot plots of astrocytic cell lysates after 24 h of *p*‐Cresol treatment at concentrations of 50, 150, or 300 μM, compared to Ctrl; reference coordinates are shown on the left. (B) Quantification of released protein (% of Ctrl) from the mixed astrocytic‐microglial medium after 24 h of *p*‐Cresol treatment. (C) Quantification of protein production (% of Ctrl) in astrocytic‐microglial cell lysates after 24 h of *p‐*Cresol treatment. (D) Quantification of released protein (% of Ctrl) in the astrocytic‐only medium after 24 h of *p‐*Cresol treatment. (E) Effects of *p*‐Cresol treatment on IL6 concentration in BV2 microglial or DINTC1 astrocytic medium measured by ELISA test. All experiments were conducted in duplicates and are displayed as mean ± SEM values. One‐way ANOVA or Student's *t* tests were performed.

To ascertain whether the increase or decrease in mRNA expression of targeted genes was accompanied by changes at the protein level, a proteome profiler kit mapping 29 chemokine/cytokine and inflammatory‐related proteins was used (Figure 7 and Table 3). Conditioned medium was collected from Ctrl and 150 μM *p*‐Cresol‐treated BV2 microglia and DITNC1 astrocytes (Figure 7B), as well as cell lysate, to assess the intracellular presence of those proteins (Figure 7C,D). Analysis from astrocyte medium showed a significant increase in some factors such as cytokine‐induced neutrophil chemoattractant 1 (CINC‐1), intercellular adhesion molecule 1 (ICAM‐1), LPS‐induced CXC chemokine (LIX), and TIMP metallopeptidase inhibitor 1 (TIMP‐1), and a decrease in vascular endothelial growth factor (VEGF); moreover, interleukin‐1 receptor antagonist (IL1ra), CCL3, and C‐X‐C motif ligand 7 (CXCL7) were detected in a microglia‐astrocyte mixed medium. Mapping of protein lysates of microglia/astrocyte mix showed significant elevation in CCL3.

**TABLE 3:** Summary heatmap of over (green) or under‐ (pink) production and/or release of cytokine/chemokine and inflammatory response mediators in astrocytic medium (Ctrl, 50, 150, or 300 μM *p*‐Cresol), astrocytes/microglia medium (Ctrl or 150 μM *p*‐Cresol), and astrocytes/microglia cell lysates (Ctrl or 150 μM *p*‐Cresol) after 24 h of treatment. One‐way ANOVA followed by post hoc Tukey's multiple comparisons was performed in astrocytes medium group; student's *t*‐test was performed in astrocytes/microglia medium and astrocytes/microglia cell lysate assay.

Reference number in array	Target	Microglia + astrocytes	Microglia + astrocytes	Astrocytic		300 μΜ
		Medium	Cell lysate	Medium		
		150 μM	150 μM	50 μM	150 μΜ	
A3, A4	CINC‐1	*	#	*	*	*
A5, A6	CINC‐2α/β	N/A	N/A	N/A	N/A	N/A
A7, A8	CINC3	N/A	N/A	N/A	N/A	N/A
A9, A10	CNTF	N/A	N/A	N/A	N/A	N/A
A11, A12	CX3CL1	N/A	N/A	N/A	N/A	N/A
A13, A14	GM‐CSF	N/A	N/A	N/A	N/A	N/A
A15, A16	ICAM‐1	**	**	*	**	***
					+	+++
						$$$
A17, A18	IFN‐γ	N/A	N/A	N/A	N/A	N/A
B3, B4	IL1α	N/A	N/A	N/A	N/A	N/A
B5, B6	IL1β	N/A	N/A	N/A	N/A	N/A
B7, B8	IL1ra	***	**	N/A	N/A	N/A
B9, B10	IL2	N/A	N/A	N/A	N/A	N/A
B11, B12	IL3	N/A	N/A	N/A	N/A	N/A
B13, B14	IL4	N/A	N/A	N/A	N/A	N/A
B15, B16	IL6	N/A	N/A	N/A	N/A	N/A
B17, B18	IL10	N/A	N/A	N/A	N/A	N/A
C3, C4	IL13	N/A	N/A	N/A	N/A	N/A
C5, C6	IL17	N/A	N/A	N/A	N/A	N/A
C7, C8	CXCL10	*	*	ns	ns	**
						+
						$
C9, C10	LIX	*	*	***	***	***
						$
C11, C12	L‐Selectin	N/A	N/A	N/A	N/A	N/A
C13, C14	CXCL9	N/A	N/A	N/A	N/A	N/A
C15, C16	CCL3	**	***	N/A	N/A	N/A
C17, C18	CCL20	ns	N/A	N/A	N/A	N/A
D3, D4	CCL5	ns	N/A	N/A	N/A	N/A
D5, D6	CXCL7	*	N/A	N/A	N/A	N/A
D7, D8	TIMP‐1	ns	**	#	ns	#
D9, D10	TNF‐α	N/A	N/A	N/A	N/A	N/A
D11, D12	VEGF	*	ns	ns	**	#
					++	#
						$

*Note:* *p < 0.05, ***p < 0.001, ****p < 0.0001, #*p* < 0.1 compared to Ctrl; +*p* < 0.05, ++*p* < 0.01, ++++*p* < 0.0001, #*p* < 0.1 compared to *p*‐Cresol 50 μM; $*p* < 0.05, $$$*p* < 0.001. All groups were in duplicate and shown as mean ± SEM values.

An ELISA assay was performed to detect IL6 in astrocytic or microglial medium. No difference was observed in microglial medium (one‐way ANOVA: F (2,8) = 1.062, *p* = 0.403) in both 50 and 150 μM conditions compared to Ctrl; in astrocytic medium, a significant increase in IL6 was observed in 300 μM *p‐*Cresol‐treated cells (*t*‐test: *p* = 0.008) (Figure 7E).

## 4 Discussion

Recent findings suggest that neurodevelopmental disorders are characterized by so called synaptopathies due to the convergence of genetic risk factors onto molecular pathways specifically localized at the synapse (Lepeta et al. 2016; Matteoli et al. 2023). In the first set of experiments, we focused on the effects of *p*‐Cresol exposure during the middle stage of neuronal growth, namely from DIV8 to DIV14, i.e., between the start and completion of synaptogenesis (Dotti et al. 1988). Rat hippocampal neurons were exposed to *p‐*Cresol, and the development of dendritic branching and synaptic markers was assessed using immunofluorescence staining and confocal analysis (Figure 1). Present data indicate that the exposure to *p*‐Cresol during in vitro synaptogenesis induced a reduction in excitatory synaptic markers, as shown by the significant reduction of VGLUT^+^ and PSD95^+^ excitatory synaptic markers, with maintenance of inhibitory synapses. In addition, we also found a decrease in the total dendrite length and their arborization, as demonstrated by the significant reduction in secondary dendrite number. Thus, taken together, these data confirm a previous study on *p*‐Cresol effects on neurons in vitro (Guzman‐Salas et al. 2022) and tally well with previous studies demonstrating a correlation between dendritic arborization and excitatory synaptic strength (Peng et al. 2009), both in vitro and in vivo (Kraeuter et al. 2019; Lin and Koleske 2010).

Then, we questioned whether *p*‐Cresol toxicity during synaptogenesis could be influenced by known ASD‐related genetic factors (Zhuang et al. 2024). Specifically, the *Shank3* gene encodes for a scaffolding protein required for correct synaptic assembly, and its variants are linked to ASD in both humans and transgenic mouse models (Varghese et al. 2017; Uchino and Waga 2013; Durand et al. 2007). Here, we demonstrated that a reduction in Shank3 protein by about 30% potentiates the effect of *p*‐Cresol on VGLUT expression and total dendritic length. This reduction was assessed at the single‐cell level by quantifying SHANK3 IR specifically in GFP‐positive neurons, i.e., successfully transduced cells. Notably, all analyses were restricted to GFP‐positive neurons, ensuring that only successfully transduced cells were included in the quantification. This approach allows direct comparison between control and knockdown neurons while avoiding signal dilution from non‐transduced cells, a limitation inherent to bulk biochemical assays such as western blotting in mixed cultures. We acknowledge that this represents a partial knockdown, which is consistent with the moderate reduction observed in synaptic puncta and aligns with previous studies showing that even partial SHANK3 reduction can lead to measurable synaptic alterations. On the other hand, VGAT expression remains unchanged in all experimental groups, suggesting that *p*‐Cresol treatment primarily affects excitatory synapses in both wild‐type and genetically susceptible backgrounds (Figure 2).

For these experiments we used commercial neuronal cultures that contain non‐neuronal cells. Mixed hippocampal cultures intrinsically include glial populations, primarily astrocytes and, to a lesser extent, microglia and oligodendrocyte lineage cells, which contribute to neuronal homeostasis and are widely recognized as a standard feature of this experimental model. Although we did not perform a direct quantitative characterization of non‐neuronal subtypes in the present study, we acknowledge this as a limitation. Nevertheless, the use of mixed cultures provides a more physiologically relevant environment, where neuron–glia interactions can modulate neuronal responses to external stimuli. While previous studies using neuronal cell lines have characterized the direct toxic effects on neurons alone, in the present study we aimed to determine whether these effects are also modulated by non‐neuronal cells (e.g., astrocytes and microglia), which may enhance or modify neuronal responses (Guzman‐Salas et al. 2022). Yet, we underline that the analytic methods we have used, being based on morphological and immunofluorescence approaches, allow us to attribute the observed changes elicited by *p*‐Cresol to the population of neurons present in the culture, although indirect contributions from glial cells cannot be excluded.

Indeed, beyond genetics, inflammation is a well‐recognized environmental risk factor for synaptopathy, the so‐called immune‐synaptopathies, such as ASD and Phelan‐McDermid syndrome (Asta et al. 2024; Sacco et al. 2012; Persico et al. 2012). Increasing evidence demonstrates that several immune system molecules critically contribute to synaptic instability and dysfunction during both highly sensitive pre‐ and peri‐natal stages, thus leading to long‐lasting neurological consequences during adulthood (Smith et al. 2007; Nordahl et al. 2013; Piras et al. 2014; Kim et al. 2023).

Here, we treated microglia and astrocytes with *p*‐Cresol for 24 h at 50, 150, or 300 μM concentration (Figures 3 and 4). Cell counting data show that microglia are much more sensitive to *p*‐Cresol treatment than astrocytes, especially at the highest *p*‐Cresol concentration, where microglia are reduced by about 70%, whereas astrocytes remain unaltered for all concentrations. The decrease in cell number is accompanied by a strong cell activation in microglia, measured as CellROX^+^ area, which is significantly increased at 50 μM, peaks at 150 μM, and then decreases at 300 μM, probably because of excessive toxicity; in astrocytes, CellROX^+^ area is increased at 150 μM and becomes even higher at 300 μM, without changes in cell number. These data indicate that *p*‐Cresol induces oxidative stress in astrocytes and microglia, producing ROS and cell‐activation. Specifically, microglia activation is confirmed by *CD11b* upregulation (Roy et al. 2006) accompanied by endolysosomal dysfunction as demonstrated by *CST7* overexpression (Daniels et al. 2023). Differences in responses to *p*‐Cresol treatment in term of ROS production might be due to intrinsically differential activation properties of the two cell lines. Accordingly, a previous study suggests that microglia and astrocytes have different sensitivity to external toxicants, and a different timing of adaptive responses (Ni et al. 2011). Clinical evidence from ASD children and *postmortem* samples shows that glial activation is often associated with cytokine and chemokine upregulation (Vargas et al. 2005; Inga Jacome et al. 2016). Our qPCR analysis demonstrated a cell‐specific induction of gene expression as a consequence of *p*‐Cresol exposure (Figures 5 and 6); specifically, *IL1β*, *IL4*, *ΤΝFa*, *CCL3*, *CCL6* increased in microglia, and *TNFa*, *IL6*, *IL18*, and *IL12* in astrocytes. Of note, all these genes are involved in the function or stability of neurons or synapses and are frequently reported in clinical, *post mortem*, and in vitro studies of ASD patients or animal models (Voineagu et al. 2011; Garbett et al. 2008; Andrews et al. 2017; Arenella et al. 2022).

The effects of IL1β elevation on synapse integrity have been well described; for example, the exposure of primary neurons to IL1β results in synapse loss and synaptic vesicle protein downregulation, dendritic spine disruption, perturbance of synaptic architecture and plasticity. However, IL1β secreted by activated microglia suppresses GABAergic inhibitory transmission, with limited effects on glutamatergic function, therefore altering E/I balance and impairing synaptic function; excess of IL1β disrupts BDNF production and signaling, which is crucial for neuron survival and development (Patterson 2015). In addition, IL1β can impair synaptic function, an effect that is prevented by IL1ra (Mishra et al. 2012); interestingly, we have found IL1ra increase in the microglia/astrocytes mixed medium and cell lysates, but not in astrocytic medium, suggesting that its production is correlated with IL1β production in BV2 cells, possibly to mitigate the harmful effects of IL1β.

Prenatal exposure to increased levels of IL6 induces behavioral deficits in adult rodents and in non‐human primate models, and alters brain connectivity and working memory in human newborns (Wei et al. 2012); in addition, in vitro and in vivo studies demonstrated that IL6 is involved in several cellular processes, such as energy homeostasis, cell adhesion and migration (Wei et al. 2012), adult neurogenesis and axonal regeneration, and regulates cellular and molecular pathways that are specifically associated with neuronal function. For example, exposure to IL6 inhibits neurotransmitter release and reduces synapse density or enhances expression of synaptophysin and VGLUT, promoting excitatory synapse formation, depending on the source, concentration, and brain‐injected area (Gruol 2015). Also, the ICAM‐1 expression is strongly associated with the IL6 expression. IL6 interacts with the IL6 receptor and activates activating protein 1, which in turn activates ICAM‐1 expression and contributes to migration in osteosarcoma cells (Lin et al. 2013). In very recent work, increased plasma levels of ICAM‐1 were found in ASD patients, together with mRNA upregulation in the brain (Szabo et al. 2024). Accordingly, we found increased production and release (i.e., indeed dose‐dependent) of ICAM‐1 from astrocyte‐microglia mixed cell lysate and collected medium after *p*‐Cresol treatment (Figure 7); it is probably linked to the IL6 overproduction from astrocytes as shown in the present study. Szabo et al. also reported increased plasma levels of IL18, and IL1β and activity of NLRP3 in peripheral blood mononuclear cells of ASD patients (Szabo et al. 2024); all these molecules were increased in our astrocytes/microglia after *p*‐Cresol treatment as well. Another interesting molecule is CXCL10, whose gene expression was downregulated in microglia but whose protein levels were increased in mixed cell lysate and mixed or astrocytic medium. CXCL10 has pro‐inflammatory and anti‐angiogenic properties, and its higher level corresponds to an anti‐angiogenic state (Gotsch et al. 2007). VEGF is responsible for angiogenesis in the brain and promotes neuronal migration, survival, and axon guidance (Shim and Madsen 2018; Mackenzie and Ruhrberg 2012). Our data show reduced intracellular and released levels of VEGF, suggesting that *p*‐Cresol treatment could reduce angiogenetic factor production from glial cells and impair normal neuron maturation. VEGF expression is also regulated by CCL3, which is a downstream target of IL1β (Lu et al. 2003), and we found it to be upregulated intracellularly but decreased as released form. CCL3, if bound to its receptor, can enhance expression of VEGF (Luna et al. 2017; Liao et al. 2016). We speculate that *p*‐Cresol induced complex pro‐ and anti‐inflammatory responses in glial cells that attempt to maintain cellular homeostasis.

In addition to oxidative stress, viability differences and inflammatory activation patterns, *p*‐Cresol distinctly affected glial motility. Specifically, astrocytes displayed a marked reduction in migration despite maintained proliferation, whereas microglia showed decreased cell viability but relatively preserved migratory behavior. This dissociation suggests that *p*‐Cresol impacts different cellular pathways in each glial population: in astrocytes, impaired motility may result from IL6‐driven ICAM‐1 upregulation and reduced VEGF signaling, which together increase adhesion and limit cytoskeletal remodeling; in contrast, in microglia, elevated ROS and pro‐inflammatory activation likely compromise survival rather than migratory capacity. Such cell type–specific alterations in proliferation and motility highlight how *p*‐Cresol disrupts the coordinated glial dynamics required for proper synaptic development and maintenance: other than direct effects on glia (proliferation, viability) and neurons (structural impairment), *p*‐Cresol‐induced oxidative stress and cytokine release from glia, particularly IL1β and IL6, could exacerbate dendritic simplification and excitatory synapse loss observed in neurons; altered chemokines production could further disrupt glia–neuron interaction by impairing migration ability of glial cells. This bidirectional crosstalk highlights how environmental toxicants can simultaneously target both synaptic and immune signaling networks, amplifying neurodevelopmental vulnerability.

Finally, our findings should also be interpreted in light of potential differences in *p*‐Cresol sensitivity among neural cell types. Neurons, which rely heavily on oxidative phosphorylation and possess limited antioxidant reserves, are particularly vulnerable to oxidative and mitochondrial stress, two key cellular effects previously associated with *p*‐cresol exposure (Guzman‐Salas et al. 2022). In our experiments, even moderate concentrations of *p*‐Cresol altered neuronal viability and morphology, consistent with this vulnerability. In contrast, glial populations such as DINCT astrocyte‐like cells and BV2 microglia displayed greater resistance at lower concentrations but responded at higher doses with changes in inflammatory/stress‐related markers. This aligns with evidence that *p*‐Cresol (as well as its metabolite p‐cresol sulfate) can modulate glial immune signaling, including reduced ADAM10/17 activity and altered cytokine production in microglia (Zheng et al. 2022). Together, these findings suggest that neurons and glia differ not only in their sensitivity thresholds but also in their response mechanisms. Such differential susceptibility could contribute to the complex neurobiological outcomes associated with elevated *p*‐Cresol in ASD, where both neuronal dysfunction and glial activation have been reported.

Additional limitations include the use of mixed neuronal cultures without direct quantification of non‐neuronal subtypes and the assessment of SHANK3 knockdown at the single‐cell level rather than by bulk biochemical methods. These constraints are inherent to the experimental system but were mitigated by complementary approaches and careful interpretation of the data.

## 5 Conclusions

In conclusion, our in vitro data show that *p*‐Cresol reduces dendritic arborization and length, probably because of E/I imbalance in neural circuitry, as excitatory synaptic proteins significantly decrease after *p*‐Cresol treatment. Furthermore, *p*‐Cresol induces microglia and astrocyte activation and ROS production, with dysregulation or abnormal production and release of inflammatory cytokines, chemokines, and related mediators of inflammatory response. Most of these molecules are involved in neurodevelopment, neuronal maturation, axon guidance, and E/I balance, suggesting that in a more complex system like co‐culture, organoid, or rodents, the effects of *p*‐Cresol on glial cells could be additive to those on neurons, leading to dendritic collapse and synaptic dysregulation, and ultimately resulting in ASD‐like pathology and behaviors.

Taken together, our findings suggest that *p*‐Cresol may influence cellular mechanisms (e.g., mitochondrial stress, altered redox balance, oxidative stress and immune dysregulation) that have been associated with ASD pathology (Flynn et al. 2025). Our in vitro models do not recapitulate the full complexity of ASD (e.g., in vivo circuitry, behavior, genetic background). Hence we interpret our results as supporting mechanistic plausibility rather than proving causation.

## Author Contributions

**Andreas M. Grabrucker:** conceptualization, investigation, writing – review and editing. **Wenjie Liao:** investigation, writing – original draft, methodology, writing – review and editing, formal analysis, data curation. **Federica Silvestri:** data curation, formal analysis, methodology, writing – review and editing, investigation. **Monica Piemontese:** investigation, methodology, writing – review and editing, formal analysis, data curation. **Eleonora Daini:** writing – review and editing, data curation. **Martina Bodria:** writing – review and editing, data curation. **Michele Zoli:** writing – review and editing, supervision, resources, conceptualization, formal analysis. **Antonio M. Persico:** writing – review and editing. **Antonietta Vilella:** conceptualization, investigation, funding acquisition, writing – original draft, methodology, validation, writing – review and editing, visualization, software, formal analysis, data curation, supervision, resources. **Kristy Antonioni:** investigation, methodology, writing – review and editing, data curation, formal analysis.

## Funding

This study was supported by Unimore‐FAR (Competitive projects) to A.V. and MIUR Dipartimenti di Eccellenza 2018–2022 to A.V., E.D., and M.B.

## Ethics Statement

The authors have nothing to report.

## Conflicts of Interest

The authors declare no conflicts of interest.

## Supporting information


**Figure S1:** Effects of *p‐*Cresol treatment on neuronal culture. (A) Quantitative results of DAPI^+^ cells and MAP2+ neuronal cell count 24 h after *p*‐Cresol treatment (*n* = 3 independent experiments). (B) Percentage of MAP2+/DAPI+ cell after *p*‐Cresol treatment. Results were expressed as mean ± SEM. Statistical analyses were performed using the two‐way ANOVA; ***p < 0.001; +*p* < 0.05 vs. *p‐*Cres 100 μM; +++*p* < 0.001 vs. *p‐*Cres 100 μM.
**Figure S2:** Effects of *p‐*Cresol treatment removal on BV2 and DITNC1 cells. (A) Quantitative results of DAPI^+^ microglia and astrocytes cell count 24 h after p‐Cresol treatment removal (*n* = 3 independent experiments). (B) Effects of *p‐*Cresol removal on microglia proliferative capability. Representative images of proliferant microglia (red arrows) are shown in (C). Results were expressed as mean ± SEM. Statistical analyses were performed using the two‐way ANOVA; *p < 0.05; ***p < 0.001.
**Figure S3:** Effects of *p‐*Cresol treatment removal on BV2 gene expression. BV2 cells were treated with vehicle, 50 or 150 μM of *p‐*Cresol for 24 h, then the medium was changed. Twenty‐four hours later, cells were pelleted, total RNA was extracted, and qPCR was conducted. (A) Data are presented as fold change normalized over the Ctrl group mean and calibrated over RPS29/RPL27 housekeeping genes. The experimental conditions were tested in triplicate, and data were expressed as mean ± SEM and analyzed by two‐way ANOVA (B). Colorimetric scale was used to show up‐ (green) and down‐ (red) regulated genes; *p < 0.05; **p < 0.01; ***p < 0.001; $$$*p* < 0.001 vs. *p‐*Cres 50 μM; #0.10 > *p* > 0.05.
**Figure S4:** Effects of *p‐*Cresol treatment removal on DITNC1 gene expression. DINTC1 cells were treated with vehicle, 50 or 150 μM of *p‐*Cresol for 24 h, then the medium was changed. Twenty‐four hour later, cells were pelleted, total RNA was extracted, and qPCR was conducted. (A) Data are presented as fold change normalized over the Ctrl group mean and calibrated over RPS29/RPL27 housekeeping genes. The experimental conditions were tested in triplicate, and data were expressed as mean ± SEM and analyzed by one‐way ANOVA (B). The colorimetric scale was used to show up‐ (green) and down—(red) regulated genes; *p < 0.05 vs. Ctrl; $$*p* < 0.01 vs. *p‐*Cres 50 μM; #0.10 > *p* > 0.05.
**Figure S5:** Qualitative aspects of BV2‐ and DITNC1‐ cell migration after *p‐*Cresol treatment. 90% confluent microglial cells were incubated with vehicle, 50, 150, or 300 μM *p*‐Cresol, and imaged with optical microscope 4× at 0 (A–D), 6 (A.1–D.1), 24 (A.2–D.2), and 48 h (A.3–D.3) after applying scratch in the corresponding well. 90% confluent astrocytes were incubated with vehicle, 50, 150, or 300 μM *p*‐Cresol, and imaged with optical microscope 4× at 0 (E–H), 6 (E.1–H.1), 24 (E.2–H.2), or 48 h (E.3–H.3) after applying scratch in the corresponding well.

**Data S1:** jnc70457‐sup‐0002‐DataS1.xlsx.

## Data Availability

Data are contained within the article and Supporting Information.
