# Supplementary material for: Unveiling the Molecular Mechanism of Intestinal Metabolite para‐Cresol in Modulating Neuroinflammation and Synaptic Dysfunction: Implications for Autism Spectrum Disorder
Source: J Neurochem. 2026 May 15;170:e70457. doi: 10.1111/jnc.70457 (PMC13178205; doi:10.1111/jnc.70457)
Supplement: Supplementary file 1 — Figure S1: Effects of p‐Cresol treatment on neuronal culture. (A) Quantitative results of DAPI+ cells and MAP2+ neuronal cell count 24 h after p‐Cresol treatment (n = 3 independent experiments). (B) Percentage of MAP2+/DAPI+ cell after p‐Cresol treatment. Results were expressed as mean ± SEM. Statistical analyses were performed using the two‐way ANOVA; ***p < 0.001; +p < 0.05 vs. p‐Cres 100 μM; +++p < 0.001 vs. p‐Cres 100 μM. Figure S2: Effects of p‐Cresol treatment removal on BV2 and DITNC1 cells. (A) Quantitative results of DAPI+ microglia and astrocytes cell count 24 h after p‐Cresol treatment removal (n = 3 independent experiments). (B) Effects of p‐Cresol removal on microglia proliferative capability. Representative images of proliferant microglia (red arrows) are shown in (C). Results were expressed as mean ± SEM. Statistical analyses were performed using the two‐way ANOVA; *p < 0.05; ***p < 0.001. Figure S3: Effects of p‐Cresol treatment removal on BV2 gene expression. BV2 cells were treated with vehicle, 50 or 150 μM of p‐Cresol for 24 h, then the medium was changed. Twenty‐four hours later, cells were pelleted, total RNA was extracted, and qPCR was conducted. (A) Data are presented as fold change normalized over the Ctrl group mean and calibrated over RPS29/RPL27 housekeeping genes. The experimental conditions were tested in triplicate, and data were expressed as mean ± SEM and analyzed by two‐way ANOVA (B). Colorimetric scale was used to show up‐ (green) and down‐ (red) regulated genes; *p < 0.05; **p < 0.01; ***p < 0.001; $$$p < 0.001 vs. p‐Cres 50 μM; #0.10 > p > 0.05. Figure S4: Effects of p‐Cresol treatment removal on DITNC1 gene expression. DINTC1 cells were treated with vehicle, 50 or 150 μM of p‐Cresol for 24 h, then the medium was changed. Twenty‐four hour later, cells were pelleted, total RNA was extracted, and qPCR was conducted. (A) Data are presented as fold change normalized over the Ctrl group mean and calibrated over RPS29/RPL27 housekeeping [file JNC-170-e70457-s001.zip › jnc70457-sup-0002-FigureS1-S5@Supplementary data_Liao et al_2026.pdf]

Unveiling the molecular mechanism of intestinal metabolite *para*-cresol in modulating neuroinflammation and synaptic dysfunction: implications for autism spectrum disorder.

Wenjie Liao[1], Kristy Antonioni[1#], Federica Silvestri[1#], Monica Piemontese[1,6], Martina Bodria[1], Eleonora Daini[1], Antonio M. Persico[1,2], Michele Zoli[1], Andreas M. Grabrucker[3,4,5*], Antonietta Vilella[1*]

1. Department of Biomedical, Metabolic and Neural Sciences, University of Modena and Reggio Emilia, 41125 Modena, Italy.
2. Child & Adolescent Neuropsychiatry Program, Modena University Hospital, Modena, Italy.
3. Department of Biological Sciences, University of Limerick, Limerick, V94PH61, Ireland.
4. Bernal Institute, University of Limerick, Limerick, V94PH61, Ireland
5. Health Research Institute (HRI), University of Limerick, Limerick, V94PH61, Ireland
6. International School of Advanced Studies, University of Camerino, 62032 Camerino, Italy

# equal contribution

*equal contribution

Supplementary data

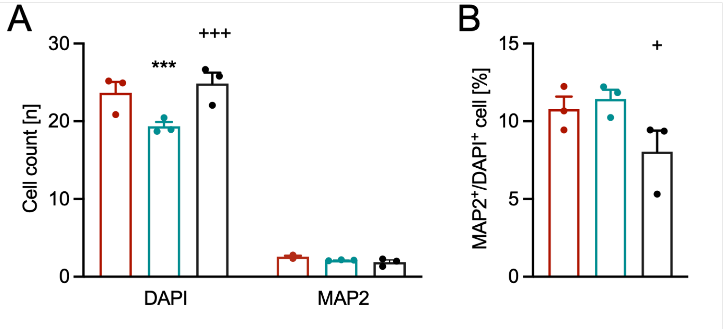

Supplementary Figure 1: Effects of *p*-Cresol treatment on neuronal culture. (A) Quantitative results of DAPI$^+$ cells and MAP2+ neuronal cell count 24 hours after p-Cresol treatment (n = 3 independent experiments). **(B)** Percentage of MAP2+/DAPI+ cell after *p*-Cresol treatment. Results were expressed as mean ± SEM. Statistical analyses were performed using the two-way ANOVA; *** p <0.001**;** + p < 0.05 vs *p*-Cres 100 µM; +++ p < 0.001 vs *p*-Cres 100 µM.

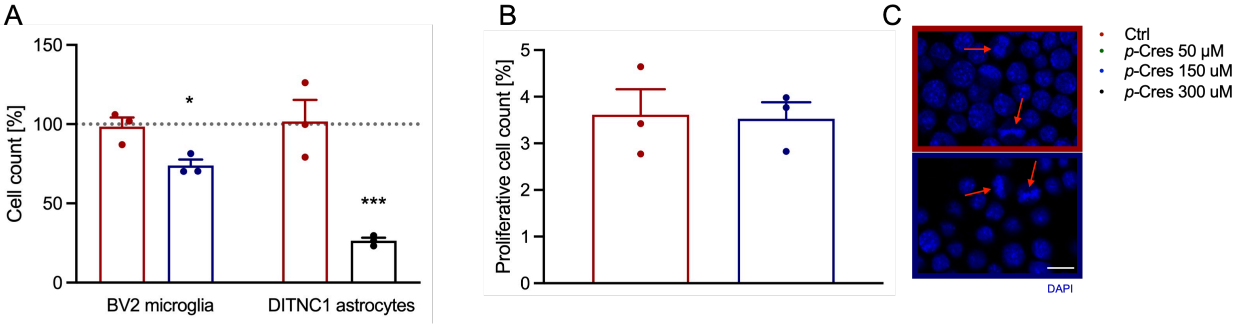

Supplementary Figure 2: Effects of *p*-Cresol treatment removal on BV2 and DITNC1 cells. (A) Quantitative results of DAPI^{+} microglia and astrocytes cell count 24 hours after p-Cresol treatment removal (n = 3 independent experiments). **(B)** Effects of *p*-Cresol removal on microglia proliferative capability. Representative images of proliferant microglia (red arrows) are shown in **(C)**. Results were expressed as mean ± SEM. Statistical analyses were performed using the two-way ANOVA; * $p < 0.05$; *** $p < 0.001$**.**

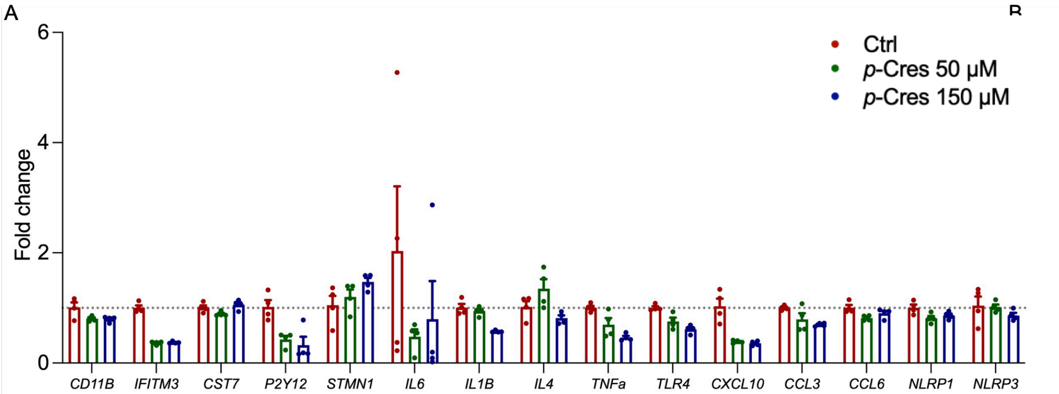

Target	50 µM	150 µM
CD11B	#	#
IFITM3	***	***
CST7	ns	ns
P2Y12	*	**
STMN1	ns	ns
IL6	ns	ns
IL1B	ns	***, $$$
IL4	ns	ns
TNFa	*	**
TLR4	*	***
CXCL10	***	***
CCL3	ns	*
CCL6	*	ns
NLRP1	*	ns
NLRP3	ns	ns

Supplementary Figure 3: Effects of *p*-Cresol treatment removal on BV2 gene expression. BV2 cells were treated with vehicle, 50 or 150 µM of *p*-Cresol for 24 hours, then the medium was changed. Twenty-four hours later, cells were pelleted, total RNA was extracted, and qPCR was conducted. **(A)** Data are presented as fold change normalized over the Ctrl group mean and calibrated over RPS29/RPL27 housekeeping genes. The experimental conditions were tested in triplicate, and data were expressed as mean ± SEM and analyzed by two-way ANOVA **(B).** Colorimetric scale was used to show up- (green) and down- (red) regulated genes; * $p < 0.05$; ** $p < 0.01$; *** $p < 0.001$; $$$ $p < 0.001$ vs *p*-Cres 50 µM; # $0.10 > p > 0.05$.

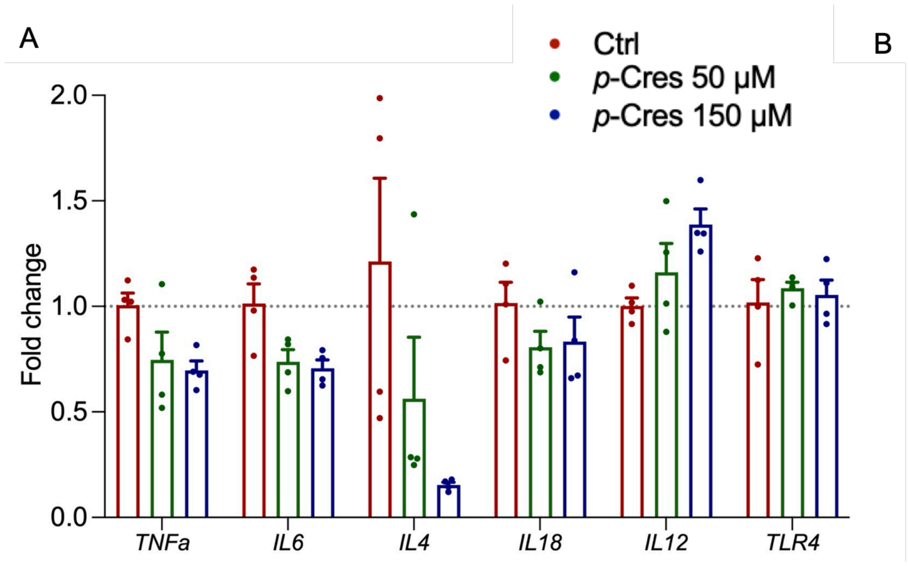

B

Target	50 µM	150 µM
TNFa	ns	#
IL6	*	*
IL4	ns	#
IL18	#	ns
IL12	ns	*,$$
TLR4	ns	ns

Supplementary Figure 4: Effects of *p*-Cresol treatment removal on DITNC1 gene expression. DINTC1 cells were treated with vehicle, 50 or 150 µM of *p*-Cresol for 24 hours, then the medium was changed. Twenty-four hour later, cells were pelleted, total RNA was extracted, and qPCR was conducted. **(A)** Data are presented as fold change normalized over the Ctrl group mean and calibrated over RPS29/RPL27 housekeeping genes. The experimental conditions were tested in triplicate, and data were expressed as mean ± SEM and analyzed by one-way ANOVA **(B).** The colorimetric scale was used to show up- (green) and down-- (red) regulated genes; * $p < 0.05$ vs. Ctrl; $$ $p < 0.01$ vs. *p*-Cres 50 µM; # $0.10 > p > 0.05$.

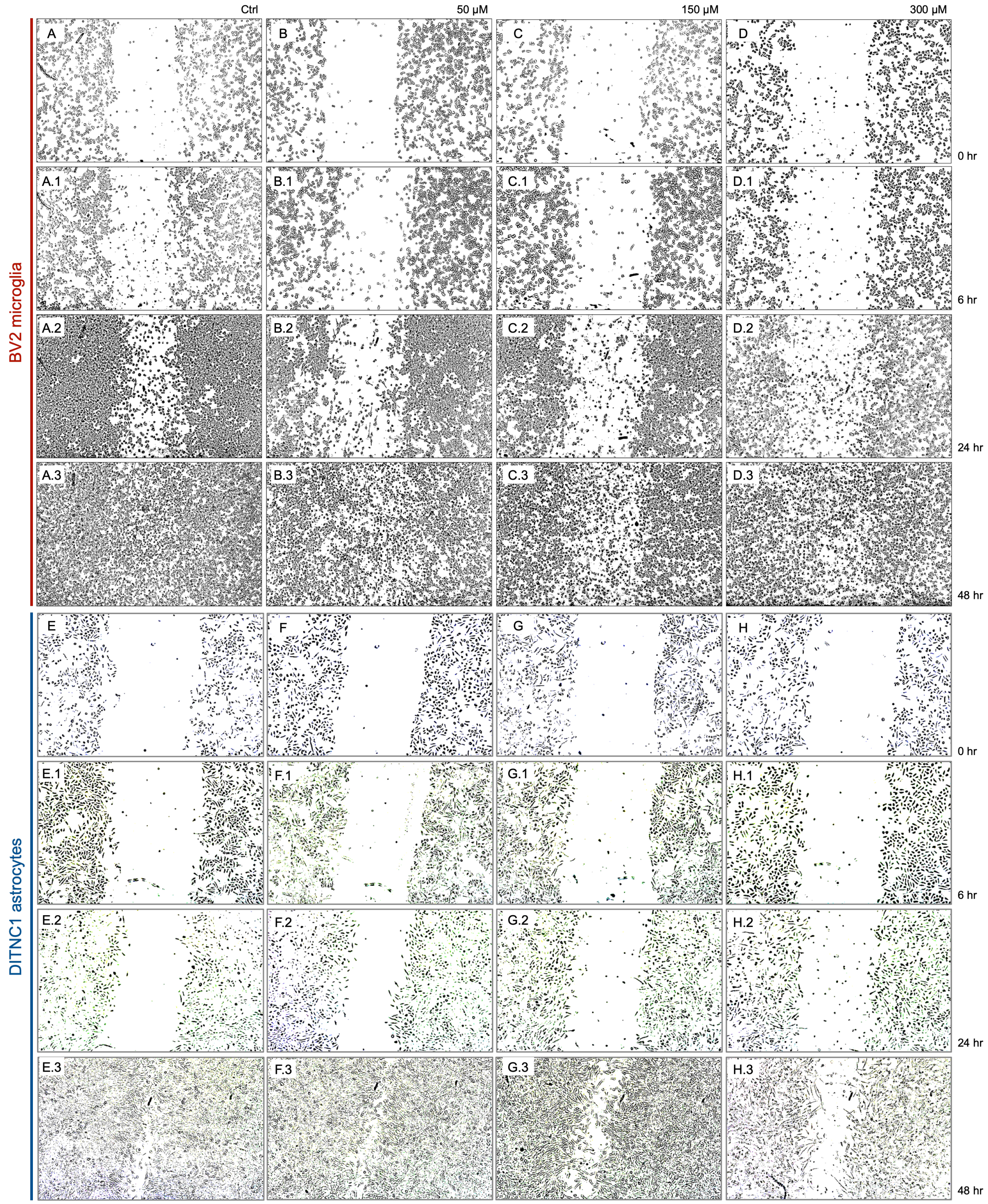

Supplementary Figure 5: Qualitative aspects of BV2- and DITNC1- cell migration after *p*-Cresol treatment. 90% confluent microglial cells were incubated with vehicle, 50, 150, or 300 µM *p*-Cresol, and imaged with optical microscope 4x at 0 (**A-D**), 6 (**A.1-D.1**), 24 (**A.2-D.2**), and 48 hours (**A.3-D.3**) after applying scratch in the corresponding well. 90% confluent astrocytes were incubated with vehicle, 50, 150, or 300 µM *p*-Cresol, and imaged with optical microscope 4x at 0 (**E-H**), 6 (**E.1-H.1**), 24 (**E.2-H.2**), or 48 hours (**E.3-H.3**) after applying scratch in the corresponding well.
